# Role of Mitochondria in Neonatal Hypoxic-Ischemic Brain Injury

**Published:** 2015-12-09

**Authors:** Yujiao Lu, Donovan Tucker, Yan Dong, Ningjun Zhao, Xiaoying Zhuo, Quanguang Zhang

**Affiliations:** 1 Department of Neuroscience & Regenerative Medicine, Georgia Regents University, USA

**Keywords:** Hypoxic-ischemia, Mitochondria, Apoptosis, Neuroprotection

## Abstract

Hypoxic-ischemia (HI) causes severe brain injury in neonates. It’s one of the leading causes to neonatal death and pediatric disability, resulting in devastating consequences, emotionally and economically, to their families. A series of events happens in this process, e.g. excitatory transmitter release, extracelluar Ca^2+^ influxing, mitochondrial dysfunction, energy failure, and neuron death. There are two forms of neuron death after HI insult: necrosis and apoptosis, apoptosis being the more prevalent form. Mitochondria handle a series of oxidative reactions, and yield energy for various cellular activities including the maintainance of membrane potential and preservation of intracellular ionic homeostasis. Therefore mitochondria play a critical role in neonatal neurodegeneration following HI, and mitochondrial dysfunction is the key point in neurodegenerative evolution. Because of this, exploring effective mitochondria-based clinical strategies is crucial. Today the only efficacious clinic treatment is hypothermia. However, due to its complex management, clinical complication and autoimmune decrease, its clinical application is limited. So far, many mitochondria-based strategies have been reported neuroprotective in animal models, which offers promise on neonatal therapy. However, since their clinical effectiveness are still unclear, plenty of studies need to be continued in the future. According to recent reports, two novel strategies have been proposed: methylene blue (MB) and melatonin. Although they are still in primary stage, the underlying mechanisms indicate promising clinical applications. Every neurological therapeutic strategy has its intrinsic deficit and limited efficacy, therefore in the long run, the perfect clinical therapy for hypoxic-ischemic neonatal brain injury will be based on the combination of multiple strategies.

## Introduction

Neonatal hypoxic-ischemic brain injury is a newborn encephalopathy caused by hypoxic-ischemia (HI) and decreased blood circulation, consequently leading to the immature nerve injury.^[1, 2]^ It’s one of the leading causes for permanent children neurological impairments. According to some studies, 0.2%to 0.4% of all full-term infants sustain asphyxial injury around the time of birth,^[3]^ and about 50% of the infants suffering severe cases will die as a newborn. Even the survivors will have heavy neurological deficits during childhood, delaying their academic progress. Brain tissue edema, tissue softening, neuronal apoptosis or necrosis, as well as hemorrhage, are its main clinical performances. Depending on the severity, the newborns usually have different post-ischemic impairments, such as cerebral palsy, epilepsy, deafness, blindness, or even death.

HI can induce a line of injurious events: release of excitatory amino acids, usually glutamate; opening of NMDA-type glutamate receptor-operated channels; intracellular ca^2+^ accumulation; mitochondria dysfunction, and mitochondrial permeabilization. The permeabilization of mitochondria will further trigger the release of cytochrome c (cyt c) and apoptosis-inducing factor (AIF), then the programmed apoptosis will start.

The only effective clinical therapy on hypoxic-ischemic neurodegeneration is hypothermia which should be performed soon after the initial insult. In neonates, many evidences from clinical trials, including tests on both whole body cooling(esophageal T=33.5°C for 72 h) and head cooling (rectal T=34–35°C, head cooling unit with water T=8–12 °C for 72 h), have shown efficacious neuroprotection. This breakthrough indicated promise in reducing the risk of disability or death after immature brain hypoxic-ischemic insult.^[4]^ However, due to the complex management, clinical complication and autoimmune decrease during hypothermia operation, its application in clinical arena has been limited to some extent. Therefore, finding other alternative therapies is urgent.

## Mitochondria and post-ischemic immature brain injury

Mitochondrial dysfunction plays a central role in HI-induced neurodegeneration, and determines the fates of cells subjected to HI.^[5–13]^ Mitochondria handle a series of oxidative reactions which produce toxic oxygen free radicals, and produce energy that is needed for various cell activities. They are also important intracellular Ca^2+^ buffers to keep intracellular homeostasis. Therefore, mitochondrial dysfunction will lead to a series of lethal events: intracellular Ca^2+^ accumulation, oxidative stress accompanied by mitochondrial energy failure, and consequent neuron apoptosis. In this brief review, we will discuss these important mechanisms that closely relate to neonatal hypoxic-ischemic neurodegeneration. (Table. 01)

## Intracellular Ca^2+^ accumulation

In normal conditions, Ca^2+^ concentration in cytosol is extremely low (about 100nM), and its extracellular concentration can reach up to 1–2 Mm.^[14]^ The intracellular Ca^2+^ concentration is maintained via several mechanisms: (1) entry of extracellular Ca^2+^ into cytosol through NMDA receptors or voltage-gated Ca^2+^ channels. (2) Ca^2+^ release from endoplasmic reticulum. (3) Ca^2+^ release through Ca^2+^-ATPase or Na^+^-Ca^2+^ exchanger in cell membrane. (4) The binding of Ca^2+^ with specific proteins. (5) Ca^2+^ transportation into mitochondria through an electrophoretic uniporter.^[15–17]^ Some studies which show NMDA antagonists can block Ca^2+^ influx into neurons, and significantly attenuate neonatal neurodegeneration after HI, indicating that the NMDA receptor channel is possibly the main pathway of intracellular Ca^2+^ accumulation.^[18]^ Under hypoxic-ischemic conditions, abnormal blood circulation deprives oxygen and glucose (DOG) supply for newborns, which subsequently triggers the overstimulation of excitatory glutamate release and the opening of NMDA receptor channels, as well as Ca^2+^ flooding into neurons.^[19]^

Elevated Ca^2+^ level in neurons can induce mitochondrial dysfunction and mediate irreversible immature neuron death by the activation of several Ca^2+^- dependent proteins. Calpains are a family of Ca^2+^- dependent cysteine proteases that decompose some vital proteins, like Ca^2+^- ATPase, protein kinase C, spectrin, and nuclear factor kappa B,^[20, 21]^ which then leads to cell remodeling, membrane destruction and neuron degeneration.^[22–24]^ Cytosolicphospholipase A2 (cPLA2) is another Ca^2+^ dependent enzyme that produces free fatty acid (e.g. arachidonic acid) and lysophospholipids by catalyzing the decomposing of glycerophospholipids, and generating superoxide.^[25, 26]^ The increased cPLA2 level in neonatal focal cerebral ischemia corresponds to its role in mediating neurodegeneraion.^[27–31]^ Nitric oxide synthase (NOS) which yields toxic free radical gas NO, and forms more toxic peroxynitrite^[32]^ is another enzyme activated by elevated Ca^2+^. Moreover, accumulated Ca^2+^ together with ROS within mitochondria contributes to mitochondrial permeability transition (MPT) and subsequent nicotinamide adenine dinucleotide (NAD) loss which is needed in various cellular energy metabolic reactions and ROS detoxification.

### Oxidative stress and metabolic failure

Reactive oxygen species (ROS) are mainly generated in mitochondria which are also the sites of ATP production by consumption of 90% of O_2_ supply. During electron transportation in mitochondrial inner membrane, the electron leakage from electron transport chain (ETC) occurs spontaneously, and the leaked electrons reacting with O_2_ will generate oxidative free species. As mentioned above, Ca^2+^ flooding into cytosol can activate the Ca^2+^- dependent enzymes, like NOS which yields toxic free radical gas NO, and forms more toxic peroxynitrite. In normal conditions, the superoxide is cleared by superoxide dismutase and glutathione peroxidase. However, during hypoxic-ischemic injury, massive of free species can’t be eliminated by antioxidant enzymes immediately due to the interrupted metabolism, leading to excessive ROS accumulation consequently.^[33]^

ROS accumulation and oxidative modifications to proteins, lipids, or DNA is the leading cause of metabolic failure and mitochondrial dysfunction following newborn hypoxic-ischemic insult. One of the oxidative target is Complex I in ETC, an enzyme that oxidizes NADH and reduces ubiquinone. Complex I is also considered as a rate-limiting protein in the process of electron transportation.^[34–37]^ The superabundant ROS and reactive nitric species (RNS) in mitochondria interrupt normal ETC function, which will in turn amplify the production of mitochondrial free species.^[38–42]^ The oxidative modifications to proteins and lipids within mitochondrial membranes alter mitochondrial inner membrane permeability, leading to membrane depolarization, which further uncouples oxidative phosphorylation processes and results in consequent failure of ATP production.^[43–45]^ This drastic energetic decrease in turn contributes to cell membrane depolarization and Ca^2+^ influx. In addition to the impairments to ETC, excessive ROS limit glucose metabolism as well. In glycolysis, the activity of pyruvate dehydrogenase complex (PDHC) will be inhibited by excessive ROS accumulation. Due to PDHC inactivity and the impairment of reducing power transportation from glycolysis-generated NADH to mitochondria, lactate dehydrogenase, which is activated by elevated Ca^2+^, will use NADH to form massive amounts of lactate in cytosol.^[46]^ The subsequent excessive lactate accumulation in neurons after HI leads to acidosis. Using magnetic resonance spectroscopy (MRS), we can see the increased lactate level in immature brains, which corresponds with the long-term studies.^[47]^

### Apoptosis

The accumulated Ca^2+^ in cytoplasm together with ROS will induce mitochondrial permeability transition (MPT), activating the opening of mitochondrial permeability transition pore (MPTP) within the mitochondria inner membrane. The role of MPTP in hypoxic-ischemic neurodegeneration has been supported by the experimental result that MPTP inhibitor cyclosporine A (CsA) could protect against HI by binding to cyclophilin D (CypD), the component of MPTP, interrupting its involvement in MPTP construction.^[48–51]^ In immature models, MPT seems more active and CypD expression in neurons is relatively high compared with mature brain. However, due to the extent of MPT involving in neonatal insult is still unclear, this CypD-based treatment for newborn post-ischemic insult is controversial.^[52, 53]^ Nicotinamide adenine dinucleotide (NAD^+^), a cofactor needed in kinds of energy metabolic reactions and ROS detoxification, will then be released through MPTP into cytoplasm. Mitochondrial distress will signal the formation of Bax/Bak megapores within mitochondria outer membrane, resulting in release of mitochondrial contents, including Cyt c and AIF. These pro-apoptosis proteins subsequently trigger the activation of cysteine-dependent aspartate-directed proteases (caspase), such as caspase 3. An irreversible apoptosis will consequently occur.^[54, 55]^ Fragmented DNA that is induced by caspase and ROS, will in turn activate poly (ADP-ribose) polymerase (PARP) which is a DNA repairing enzyme. The DNA repairing process at the same time consumes NAD^+^, affecting its concentration in mitochondria. NAD^+^ loss plus oxidative failure will worsen mitochondrial dysfunction, and extend the impairment of neonatal hypoxic-ischemic brain injury. During neurodegeneration, two forms of neuron death could happen: necrosis or apoptosis, and the final outcome depends on the severity of initial hypoxic-ischemic insult. Generally, an intense mitochondrial insult tends to produce necrosis, while a milder one is more likely to cause apoptosis.^[56]^ Plenty of previous studies have elucidated that apoptosis is the more prevalent form in immature brain injury compared with adult models.^[57, 58]^

## Mitochondria-based neuroprotective strategies in immature brain

Mitochondria play central role in energy generation which is needed for various cell activities. They also handle a series of reactions yielding toxic oxidative free species. Moreover, mitochondria are intracellular Ca^2+^ buffers, which is important for cell ionic homeostasis. Mitochondrial dysfunction, as depicted above, is the key point in neonatal neurodegeneration after hypoxic-ischemic brain insult. Therefore exploring mitochondria-based neuroprotective strategy is critical for the treatment of hypoxic-ischemic neurodegeneration. According to recent studies, several methods of different mitochondria-related therapeutic targets have been tested in animal models, and reported efficacious in post-ischemic immature brain injury, indicating promises for future clinical applications. (Table. 02)

### Bax-Inhibiting Peptide (BIP)

In the evolution of hypoxic-ischemic neurodegeneration, mitochondrial will trigger the translocation of Bax protein, a switch protein mediating cell apoptosis, and induce mitochondrial outer membrane permeabilization by forming Bax-involved megapores within mitochondrial outer membrane. The formation of Bax megapores facilitates Cyt c and AIF release from mitochondrial matrix into cytoplasm, leading to both caspase- and caspase-independent neuron death.^[59, 60]^ In this sense, the formation of Bax megapores seems to be the starting point of apoptosis. Thus inhibiting Bax’s activity may offer a promising possibility for immature neurological protection. Bax-inhibiting peptide, which is derived from Ku70 protein, is able to interact with Bax protein by the Bax-binding domain of Ku70 and the N-terminal 53 amino acids of Bax, preventing its involvement in the formation of Bax megapores, and reducing the subsequent downstream caspase activation.^[61, 62]^ According to a report from Wang et al.,^[63]^ BIP confers improvement in both sensorimotor and memory functions of newborn rats at 7 weeks after HI by early BIP injection before HI happens, which reveals remarked neurological therapeutic function after neonatal hypoxic-ischemic insult.

### Inhibition of PARP

DNA fragmentation in apoptosis can activate PARP, a DNA repairing enzyme, and consume NAD^+^ at the same time, which will limit this co-factor’s concentration in mitochondria. The decrease in NAD^+^, together with oxidative deficit leads to mitochondrial energy failure and membrane depolarization, causing a worse neurodegeneration consequently. So theoretically speaking, the inhibition of PARP can reduce the loss of NAD^+^, and preserve mitochondrial energy metabolism. In this sense, PARP can be a therapeutic target by the administration of PARP inhibitor, such as 3-aminobenzamide. Some studies have proved an excellent neuroprotection by pretreatment with 3-aminobenzamide (3-AB, 10 mg/kg) in neonatal rats, showing a strongly reduced PARP activation and neurons death,^[64]^ which raises the possibility of using PARP inhibitor as a potential method for neurological therapy after neonatal hypoxic-ischemic brain insult.

### Inhibition of MPT

Although it is still controversial about the extent MPT involves in neonatal brain injury, it has been widely accepted that MPT plays a crucial role in the mediation of post-ischemic neurodegeneration.^[65–67]^ CypD which is located in mitochondrial matrix, has been proved as an important component of MPTP within mitochondrial inner membrane by the studies that CypD knock out can effectively block MPTP formation.^[68–70]^ Thus, the inhibition of CypD could be a promising strategy for immature brain injury after HI insult. Many previous studies have proven its neuroprotection in mature models after HI by administrating CsA, a CypD inhibitor that can bind to CypD to block its involvement in MPTP formation.^[48–51]^ In immature models, according to Roman A. Eliseev et al.,^[53]^ the expression of CypD in neurons is age-dependent, it is of higher level in neonatal rat brain than adult rat brain, and CypD expression down regulates in the process of brain development. However, the effectiveness of this CypD-targeted neuroprotective strategy in immature brain is still in hot debate. More studies in this direction in the future will be informative.

### Administration of nicotinamide

Ca^2+^ accumulation within mitochondria, together with intramitochondrial ROS can trigger the opening of permeability transition pores (PTP) within mitocondrial inner membrane. The consequence of PTP opening is the loss of co-factor NAD^+^ which involves energy metabolisms, leading to further energy deficits. Prompt NAD+ replenishing after hypoxic-ischemic insult can compensate the NAD^+^ loss from mitocondria, and enhance aerobic energy metabolism. Experiments targeted at NAD+ administration in animal models have been tested, showing the administration of nicotinamide in rat model at dose of 500 mg/kg by intraperitoneal injection up to 2 h after the onset of permanent focal cerebral ischemia can significantly reduce the infarct volume,^[71]^ increase regional cerebral blood flow (rCBF) and metabolic rate of oxygen.^[72]^ Other report indicates that nicotinamide administration into neonatal rats as long as 24 h after perinatal asphyxia can preserve dopamine levels within striatum 3 months later at a relative high level.^[73, 74]^ These preclinical studies, although performed in animal models, still reveal promising outlook for neonatal post-ischemic treatment.

### Administration of ketogenic diet

Since the activity of aerobic glucose metabolic enzymes, like pyruvate dehydrogenase, are inhibited after hypoxic-ischemic insult, the energy deficit will be induced consequently. Moreover, in immature brain, the levels of vascular monocarboxylic acid transporters that carry ketone bodies and ketone body metabolic enzymes are relatively high,^[73, 75]^ ketone bodies, especially β-hydroxybutyrate, can serve as an alternative fuel for cerebral energy metabolism. β-hydroxybutyrate, a high-fat, low-carbohydrate and low-protein ketogenic diet (KD), has been proven neuroprotective in a series of rat models with acute brain injury,^[76–79]^ both for adult and newborn rats.^[80]^ Although KD has been shown efficacious in the treatment of childhood epilepsy, long-term KD administration can impair the normal brain development as well, ^[81]^ which will restrict its future clinical application in immature brain treatment.

### Co-administration of EP and IGF-I

The immature brain is more sensitive to ROS accumulation and displays different responses to therapeutic interventions under HI injury compared with adult brain.^[82–84]^ One of the underlying mechanisms is the inability of immature brain to clear accumulated ROS immediately.^[85–87]^ Enhancing the antioxidant ability of immature brain is beneficial for neuroprotection. Evidence from the study by Zhihui Rong et al.^[88]^ reveals that ethyl pyruvate(EP), a lipophilic derivative of pyruvic acid, has a more stable and more brilliant antioxidant function by early administration in neonatal rat model after HI insult. Its co-administration with insulin-like growth factor-I (IGF-I), a pleiotropic factor essential to immature neuron growth and proliferation which is administrated at a delayed time (24 and 48 h post-injury), shows a significant reduction in immature neuron death and long-term behavioral development in rat models. To verify its therapeutic efficacy, more detailed preclinical and clinical tests need to be done in the future.

### Future direction

Neonatal hypoxic-ischemic brain injury is one of the leading causes in newborn brain deficits, resulting in a series of neurological lesions. But currently there is still no mature therapeutic strategy available in clinical practice. Hypothermia is the only relatively effective treatment in clinical operation which has been studied for many years. However, because of its complex management, clinical complications and autoimmune decrease, its wide clinical application is still limited. Many other therapeutic methods, like the ones depicted above which have been proved neuroprotective in animal models, should be further tested in clinical practice before they are accepted as mature therapeutic methods. According to recent studies, two novel therapies: Methylene blue (MB) and Melatonin, show potentials for hypoxic-ischemic encephalopathy amelioration in the future.

Methylene blue which has been used in clinics for various diseases for more than a century,^[89–91]^ recently has been reported to possess neuroprotective function.^[92]^ The underlying mechanism is that MB is capable of reducing ROS production by minimizing electron leakage from the mitochondrial electron transport chain,^[93]^ promoting glucose uptake and increasing intracellular ATP concentration and O_2_ level, leading to a significant cell viability elevation.^[92]^ As a novel strategy, it is receiving more attentions to its potential for an effective clinical option in the future.

Melatonin (N-acetyl-5-methoxytryptamine) is an important neurohormone secreted by pineal gland to transduce information about light-dark cycles for body rhythms organization.^[94]^ It is considered as a direct scavenger capable of clearing a wide variety of free radicals (FR), relieving oxidative stress after neonatal HI insult.^[95–97]^ Moreover, melatonin has anti-inflammatory function by down-regulating the production of some inflammation related cytokines, like 8-isoprostanes, a potent mediator of HI induced inflammation.^[98]^ Finally, melatonin has been reported to have anti-apoptosis activity by preventing Cyt c release,^[99, 100]^ diminishing Bax^[101]^ and Bad^[102]^ pro-apoptosis proteins, and reducing DNA fragmentation.^[102]^ Although enough evidences have proven melatonin’s neuroprotective benefits in animal models, many highlighted work needs to be done before its clinical application comes true, because several of its functions are not yet elucidated.

Since every neurological therapeutic strategy, either relatively mature clinical physical tools like hypothermia, or immature pre-clinical agent study, as mentioned above, has an intrinsic deficit or limited efficacy, it will be difficult for one treatment alone to cure the post-ishemic brain injury completely. This means that the combination of multiple therapies should be the right direction in the future.

## Conclusions

Since neonatal hypoxic-ischemic brain injury can lead to a line of childhood neurological lesions, and mitochondria play central role in this pathological process, the preservation of their functional integrity should be a priority. So far, a series of therapeutic methods, as mentioned above, have been proven significantly neuro protective in animal models. However, due to lacking further efficacious proof in clinical practice, there is still a long way for their wide acceptance in human treatment. Two novel strategies, MB and melatonin, which have been applied in other fields for many years, attracted our attention for neonatal HI improvement recently years. Although their efficacy in neuroprotection are in the primary stage, the underlying mechanisms still indicates promising potentials. As every therapeutic strategy reported so far has its intrinsic deficit or limited efficacy in neurological improvement, the perfect treatment of neonatal brain injury after HI will depend on the combination of multiple strategies at least in the near future.

## Figures and Tables

**Table. 01:** Main events in post-ischemic immature brain injury

Pathological Events	Mechanisms	Consequences
Intracellular Ca^2+^ accumulation	Ca^2+^ influx into cytosol caused by excitotoxity-induced NMDA reseptors channel opening and cell membrane deplorization	Mitochondrial dysfunction triggered by activation of Ca^2+^ dependent enzymes; MPT formation induced by Ca^2+^ and ROS
Oxidative stress and metabolic failure	Inability of antioxidant enzymes; Failure of ETC and uncoupling of oxidative phosphorylation caused by mitochondrial dysfunction.	Devestating modifications to protein, lipid and DNA; Cell membrane depolarization and worse cycle of Ca^2+^ accumulation
Apoptosis	Pro-apoptosis proteins activation by the release of Cyt c and AIF from Bax/Bak megapores	Irreversible neuron death and neurodegeneration

**Table. 02:** Mitochondria- based neuroprotective strategy

Strategies	Mechanisms	Results
Bax-inhibiting peptide	Inhibition of Bax protein-involved megapores formation on within mitochondrial outer membrane	Inhibition of Cyt c and AIF release from mitochondria
Inhibition of PARP	Inhibition of NAD^+^ consumption by PARP	Preservation of mitochondrial energy metabolism
Inhibition of MPT	Administration of CypD inhibitor interrupting its involvement in MPT construction, preventing NAD^+^ release from mitochondria	Effective in mature brain, controversial efficiency in immature model
Administration of nicotinamide	Compensation of NAD^+^ loss from mitochondria	Promotion of aerobic energy metabolism
Administration of KD	Fuel replenishment for cerebral energy metabolism	Efficacious in childhood epilepsy treatment Impairment on normal brain development by long term administration
Co-administration of EP and IGF-I	Attenuation of oxidative stress within mitochondria, promoting immature neurons growth	Significant reduction in immature neuron death

## Abbreviations

HI: Hypoxic-ischemia
Ca^2+^: Calcium ion
MB: Methylene blue
NOS: Nitric oxide synthase
Cyt c: Cytochrome c
AIF: Apoptosis-inducing factor
MPT: Mitochondrial permeability transition
MPTP: Mitochondrial pore
NAD: Nicotinamide adenine dinucleotide
PARP: poly (ADP-ribose) polymerase
NO: Nitric oxide
PDHC: Pyruvate dehydrogenase complex
BIP: Bax-Inhibiting Peptide
rCBF: Regional cerebral blood flow
KD: Ketogenic diet
ETC: Electron transport chain
cPLA2: Cytosolic phospholipase A2
MRS: Magnetic resonance spectroscopy
CypD: Cyclophilin D
CsA: Cyclosporin A
EP: Ethyl pyruvate
IGF-I: Insulin-like growth factor-I
DOG: Deprive oxygen and glucose
NOS: Nitric oxide synthase
RNS: Reactive nitrogen species
ROS: Reactive oxygen species
FR: Free radicals
